# College Students' Perceptions of Worry and Parent Beliefs: Associations with Behaviors to Prevent Sun Exposure

**DOI:** 10.1155/2017/4985702

**Published:** 2017-07-19

**Authors:** Robert A. Yockey, Laura A. Nabors, Oladunni Oluwoye, Kristen Welker, Angelica M. Hardee

**Affiliations:** ^1^Department of Psychiatry and Behavioral Neuroscience and Department of Health Education and Promotion, University of Cincinnati, Cincinnati, OH 45221-0068, USA; ^2^Health Promotion and Education Program, School of Human Services, University of Cincinnati, Cincinnati, OH 45221-0068, USA; ^3^Initiative for Research and Education to Advance Community Health (IREACH), Washington State University, Spokane, WA 99210-1495, USA

## Abstract

More research is needed to understand how attitudes impact behaviors that afford sun protection. The current study examined the impact of students' perceptions of parental beliefs about sun exposure and its influence on their practiced sun protection behaviors and worry about sun exposure. Participants were college students (*N* = 462) at a large Midwestern university. They completed a survey to examine their perceptions of risks and messages about sun exposure and sun exposure behaviors. Results indicated that gender and students' perceptions of parental beliefs about sun exposure were related to sun protection behaviors and their own worry over sun exposure. Specifically, males showed lower levels of sun protection behaviors, with the exception of wearing a hat with a brim, and lower levels of worry about sun exposure compared to females. Roughly a third of our sample had a family history of skin cancer, and this variable was related to worry about sun exposure and parental beliefs. Prevention messages and interventions to reduce sun risk for college students should address risks of sun exposure as well as educating young adults about the importance of wearing sunscreen, protective clothing, and hats to improve sun protection.

## 1. Introduction

Estimates from recent reports from the American Cancer Society indicated that 9,730 people die from melanoma each year, with the greatest casualties in men, and the reported numbers are continuing to rise [1, 2]. Parallel to the rising prevalence rates are the costs of treating dermatological cancers, thus creating economic hardships for some families. Guy et al. [3] reported a 126.2% increase in annual total costs in skin cancer treatment from 2002 to 2011, making the final total cost of skin cancer treatment, in 2011, $8.1 billion. Although skin cancer is a significant public health concern, sun protection behaviors often lag behind knowledge of this threat [4]. Young adults may not engage in use of sunscreen or other sun protection behaviors, such as wearing hats or avoiding sun exposure [5]. Estimates from 2015 indicated that about 33% of adults reported wearing sunscreen with SPF 15+, 38% reported wearing protective clothing, and 39% sought shade to protect themselves from the sun [6]. Greater understanding of attitudinal factors that might motivate young adults to engage in sun protection behaviors will be important to inform prevention messages for this high risk group.

The Health Belief Model and the Protection Motivation Theory suggest that perceptions, such as people's views of social norms, influence their actions related to health prevention practices, such as wearing sunscreen and hats to protect one's skin from sun exposure [7, 8]. Research has found that adults' perceptions of social norms also may influence their health protection behaviors [9]. Reid et al. [10] proposed that perceptions about others' beliefs of the importance of a behavior may influence an individual's behavior. As such, students' perceptions toward parental beliefs about the importance of sun exposure may positively influence sun protection behaviors [11].

Researchers have reported that increased anticipatory emotion, such as worry, is related to increased health protection [9, 12]. Worrying over a potential health problem is an anticipatory emotion, which may influence protective behaviors [12, 13]. As such, worrying about sun exposure has been related to increased sun protection behaviors [10, 11, 14]. In addition to worry and parental influence, there may be differences in men's and women's sun protection behaviors. Specifically, women may engage in more sun protection behaviors and worry more about the harmful effects from sun exposure relative to men [9, 15, 16].

There were two main aims of this study. The first aim was to examine the influence of worry about sun exposure and young adults' perceptions of parent beliefs about the importance of sun exposure on their sun protection behaviors. It was hypothesized that greater worry about sun exposure would be related to reports of higher levels of sun protection behaviors. Additionally, it was anticipated that greater parents' perceived importance about sun exposure would be related to higher levels of sun protection behaviors. The second aim was to examine the influence of family history of skin cancer on worry about sun exposure and perceptions of parental beliefs about the importance of sun exposure. It was hypothesized that having a family history of skin cancer would be related to greater worry and higher levels of perceived parental beliefs about sun exposure.

## 2. Materials and Methods

### 2.1. Participants

Four hundred and sixty-two college students from a large Midwestern university participated in this study (see Table 1). Mean age was 19 years and 2 months (SD: 1 year, 5 months; age range: 16 to 33 years).

### 2.2. Measure

Questions used in the* Sun Exposure Survey* were developed after a literature review [17–21]. Health education professionals in the field provided expert review of questions in our survey. Questions about sun exposure behaviors were answered on a four-point scale (“Do not know = 0; 1 = never/rarely; 2 = sometimes; 3 = most of the time; and 4 = always”). The following questions were used to assess sun exposure behaviors: “I wear sunglasses”; “I wear sunscreen”; “I wear hats in the bright sun”; “I wear chap stick with sunscreen in it”; and “I wear a hat with a brim on it.” One question was used to examine worry—“I worry about getting too much sun on my skin.” Another question was “Has anyone in your family ever had skin cancer?” (response categories were “yes, no, and do not know”). Students then circled the members of their family who had skin cancer from the following list: “Mom, Dad, Brother, Sister, Grandpa, Grandma, Aunt, Uncle, Cousin or Other Relative.” Student views of parent beliefs were assessed with the following question: “My parents think sun exposure is a big deal” (response categories were: “yes, no, and do not know”).

### 2.3. Procedures

This study was approved by a university-based institutional review board (IRB). Participants reviewed an information form, which reviewed all informed consent procedures. The information form noted that college students who completed the* Sun Exposure Survey* for this study were providing their consent to participate. Participants independently completed the* Sun Exposure Survey* and when finished with the survey placed it in a sealed envelope or box in their college classroom.

A test-retest study was also completed to assess reliability of the survey. The sixteen students (a convenience sample of college students at the same university) participating in the test-retest study reviewed a second information form, explaining the test-retest study and procedures, which also was approved by the same university-based institutional review board. These participants completed the survey two times (retest 14 days later). They placed surveys in a sealed envelope when completing them.

### 2.4. Data Analyses

Descriptive analyses were conducted on student perceptions, sun protection behaviors, and reports about family history of skin cancer. SPSS Version 23 was used for analyses. A MANCOVA was used to examine the relationship between participant sex (male or female) and student perceptions of parent beliefs of sun exposure (yes, no, or do not know) and its effect on student report of sun protection behaviors (e.g., wearing sunscreen, chap stick with sunscreen, and wearing hats) and their worry about sun exposure. The different sun protection behaviors were considered dependent variables. Age was the covariate. Two chi-square analyses were used to examine whether having a history of skin cancer in the family influenced student worry about sun exposure and student perceptions of their parents' beliefs about sun exposure being important.

## 3. Results

Results for descriptive statistics assessing sun exposure protection behaviors and questions assessing level of worry about sun exposure are presented in Table 2.

Results indicated students were most likely to wear sunscreen sometimes, and 17% of students did not wear or rarely wore sunscreen. Twenty-eight percent never/rarely wore chap stick or lip balm with sunscreen, 37% did not wear/rarely wore hats in the bright sun, and 13% never/rarely wore sunglasses. Approximately 20% of participants were worried about sun exposure (see Table 2). Four hundred and fifty-seven students also reported their perceptions of their parents' beliefs about sun exposure being a big deal. Thirty-seven (8.1%) reported they did not know their parents' beliefs. Two hundred and ninety-five (64.6%) thought their parents believed sun exposure was a big deal, while 125 (27.4%) did not believe their parents thought sun exposure was a big deal. Mean scores and standard deviations for the questions were as follows: wearing sunglasses, M = 2.46 (SD = .90); wearing sunscreen, M = 2.25 (SD = .84); wearing hats in bright sun, 1.86, (SD = .83); wearing chap stick with sunscreen, M = 2.23 (SD = 1.08); wearing a hat with a brim, M = 1.83 (SD = .90); and worry about too much sun exposure on skin, M = 1.92 (SD = .95). In general, these scores indicated variable (e.g., “sometimes”) engagement in sun protection behaviors among college students.

A 2 × 3 (sex × parent beliefs) MANCOVA with age in years as a covariate was used to examine the influence of sex and parent beliefs on college students' report of their sun protection behaviors and worry about getting too much sun exposure. Results were significant for sex, Wilks' lambda = .882, *p* < .001, and *η*_*p*_^2^ = .118, and for parental beliefs, Wilks' lambda = .903, *p* < .001, and *η*_*p*_^2^ = .05. The results for the interaction term were not significant, and age was not a significant covariate. Results of the univariate tests for the main effect of sex of the participants, with means and standard deviations, are presented in Table 3.

Results indicated females were more likely to wear sunglasses, sunscreen, and chap stick and worry about getting too much sun on their skin. Males were more likely to wear a hat in the sun or a hat with a brim in the sun (see Table 3).

Table 4 presents results of the univariate tests for the main effect of parent beliefs on student reports of sun protection behaviors and worry about sun exposure as well as means and standard deviations and results from Tukey's follow-up tests.

Results indicated a significant difference for all of the questions, with the exception of wearing sunglasses (see Table 4). Therefore, Tukey's follow-up tests were used to examine differences between “do not know,” “yes,” and “no” answers for student perceptions of parents' beliefs about sun exposure for wearing sunscreen, hats, chap stick, and a hat with a brim and worry about getting too much sun. College students were more likely to report engaging in wearing sunscreen, hats, and chap stick with sunscreen and to worry about getting too much sun if their parents viewed sun exposure as a big deal. Means for wearing sunscreen and chap stick with sunscreen were higher for students who reported, “yes”; their parents thought sun exposure was a big deal compared to those who reported they did not know their parents' beliefs about sun exposure (see Table 4).

One hundred and fifty students (32.5%) reported that a family member had experienced skin cancer. In terms of the family member who had skin cancer, 148 of the students reported who had experienced skin cancer. Specifically, 52 (35.1%) students reported the family member was a grandparent, 30 (20.3%) reported it was a parent, 40 (27%) reported multiple family members had skin cancer, and the remaining students (*n* = 26; 17.6%) reported that another family member had experienced skin cancer.

A chi-square analysis was used to examine the relationship between student perceptions of parent beliefs that sun exposure was a big deal and whether a family member had skin cancer. The family member having had skin cancer variable was recoded, combining “do not know” and “no” answers into “no” answers compared to “yes” answers. This coding occurred so that answers indicating a family history of skin cancer could be compared to other responses provided by participants. Students' reports about parent beliefs were recoded, and “do not know” and “no” answers were combined and compared to “yes” answers (using the logic of comparing “yes” responses to all other responses). The chi-square was significant, *χ*^2^ = 13.53, *p* < .001. When students viewed parents as thinking that sun exposure was a big deal, there was more likely to be skin cancer in the family (61.4%), compared to not having skin cancer in the family (38.6%). When students reported parents as not seeing sun exposure as a big deal, there was less likely to be skin cancer in the family. Specifically, for students reporting it was not viewed as a big deal by parents, 21.7% of the students reported skin cancer in the family, while 78.3% reported that there was no skin cancer in the family.

A chi-square analysis was performed to examine the relationship between personal worry and whether a family member had skin cancer. The variable for personal worry was recoded so that “never/rarely” and “sometimes” answers were combined to indicate low worry, and “most of the time” and “always” answers were combined to indicate high worry. This was compared to a family member having skin cancer (coded as “yes” or “no”). The chi-square was significant, *χ*^2^ = 9.01, *p* = .002. At the higher level of worry, 45.4% of the participants had a family member with skin cancer (54.6% did not have a family member with skin cancer). At the lower level of worry, 29.2% of the participants had a family member with skin cancer (while 70.8 did not have a family member with skin cancer).

As mentioned, a test-retest reliability study was conducted with 16 college students (female = 10; male = 6; 14 = white; 2 = black). These students completed the measure at a two-week interval. The mean age was 19 years and 7 months (SD = 1 year and 8 months; age range = 18–25 years). Correlations for the questions examining sun exposure behaviors at Time 1 and Time 2 were as follows: wearing sunglasses, *r* = .912, *p* < .001; wearing sunscreen, *r* = .872, *p* < .001; wearing hats in the bright sun, *r* = .767, *p* < .001; wearing chap stick, *r* = .622, *p* = .01; wearing a hat with a brim, *r* = .629, *p* = .009; and worry about getting too much sun on my skin, *r* = .334, *p* = .205. Kendall's tau-b for answers about parents believing sun exposure was a big deal at Time 1 and Time 2 were significant, *τ* = 2.75, *p* = .006.

## 4. Discussion

Study results were consistent with other researches indicating that adults may neglect sun protection behaviors [4–6]. Over half of our sample reported low frequencies (“never/rarely”, “sometimes”) for participating in behaviors like wearing sunglasses, sunscreen, hats, and lip balm with SPF protection. Students who mentioned that sun exposure was a big deal to their parents were more likely to engage in sun protection behaviors and endorse worry about sun exposure. Almost a third of our sample reported having a family member who had experienced skin cancer. As expected, students with a higher level of worry reported that about 45% of their family members had experienced skin cancer, whereas those with lower worry reported that about 29% of their family members had experienced skin cancer. Of participants reporting greater perceptions of sun exposure being a big deal to parents, about 61% stated that a family member had experienced skin cancer, whereas participants with lower perceptions of parent beliefs about sun exposure being a big deal had fewer family members who had experienced skin cancer. This is consistent with research that has demonstrated that having skin cancer in the family may influence perceptions toward sun exposure [14].

In general, males engaged in lower sun protection behaviors, with the exception of wearing hats with a brim. College-age males in our sample may have been more likely to wear baseball caps, as a fashion accessory, compared to females. Our results support prior research, indicating that females are more likely to worry about sun exposure and engage in more sun protection behaviors compared to males [9, 15, 16]. Messages targeted at males should highlight information to increase their knowledge of greater risks for skin cancer and how sun protection behaviors, such as wearing sunscreen and lip balm with sunscreen, have the potential to substantially lower skin cancer risk. In addition, messages should include information about the benefits of sunscreen as well as staying out of the sun at peak hours, wearing protective clothing, and using hats and sunglasses [22]. Emphasizing risks of sunburns for skin cancer and emphasizing the benefits of sunscreen and protective clothing and wearing hats may be critical components of health education messaging. Thus, there is a need for the development and use of educational materials about sun exposure dangers, especially for males who may engage in lower frequencies of sun protection behaviors.

Although our findings provide insight into college students' ideas about sun protection and potential areas for prevention and intervention campaigns, several limitations should be noted. For example, the survey used for this study was a self-report survey with a cross-sectional design, and, thus, social desirability bias may limit the generalizability of findings as does the fact that attitudes were assessed at only one point in time. Moreover, physical appearance attitudes (e.g., ideals about skin colors) were not examined, and differences in sun protection behaviors and worry about sun exposure may have been influenced by a desire to achieve an ideal skin color [22] Combining “do not know” and “no” answers for the family history questions is a limitation, because some of the participants who answered “do not know” may have had a positive family history for skin cancer. Combining the “do not know” and “no” answers for the perceptions of parental beliefs has similar limitations. Moreover, only one item was used to assess key variables. Some of the effect sizes for sun protection behaviors were fairly small; but, this was consistent with other literatures [9]. It is noteworthy that only one question had the wording “bright sun,” whereas other questions were worded differently, which may have influenced participant responses. Moreover, the wording “parents think sun exposure is a big deal” could have been interpreted as encouraging sun exposure or as discouraging sun exposure. Finally, some test-retest correlations were low. It may be that responses do not remain consistent over time. It also was noteworthy that the sample size for the test-retest study was small, which may have influenced study findings. Thus, further research assessing study questions is needed.

## 5. Conclusion

Our results indicated that behaviors to prevent sun exposure in young adulthood are influenced by college students' perceptions of parental beliefs. Prevention and intervention programs such as sunscreen dispensers and messaging about sun protective behaviors in public places for this particular population decrease various risk factors associated with sun exposure. Early educational messages addressing youth perceptions may play an important role in shaping health behaviors. Parents should emphasize the use of sunscreen and products that include SPF as well as use of protective clothing and hats to reduce risk for sunburns and sun damage. Attitudes about beauty, ideal skin color, and knowledge and experience with sun burns were not assessed in this study and remain important areas for future research.

## Figures and Tables

**Table 1:** Demographic information and student responses for sun protection behaviors.

Variable	*n*	%
Sex		
Male	185	40%
Female	275	59.5%
Ethnic group		
White	359	77.7%
Black	36	7.8%
Asian	26	5.6%
Indian^*∗*^	8	1.7%
Hispanic	6	1.3%
Biracial	21	4.5%
Other	5	.01%
College level		
Freshman and sophomores	404	87.4%
Juniors and seniors	58	12.6%

*Notes*. *N* = 462. Two students did not provide information about gender and one student did not provide information about ethnicity. ^*∗*^Indian does not refer to Native American Indians, but rather those students identifying with India.

**Table 2:** Sun protection behaviors and worry reported by college students.

Area	Question	Do not know		Never/rarely		Sometimes		Most of the time		Always	
		*n*	%	*n*	%	*n*	%	*n*	%	*n*	%
Sun protection behaviors	I wear sunglasses	1	.2%	62	13.4%	187	40.6%	145	31.5%	66	14.3%
	I wear sunscreen	1	.2%	79	17.1%	221	47.8%	123	26.6%	37	8%
	I wear hats in the bright sun	1	.2%	174	37.7%	194	42.1%	74	16.1%	18	3.9%
	I wear chap stick with sunscreen in it	6	1.3%	130	28.2%	155	33.6%	92	20%	78	16.9%
	I wear a hat with a brim on it	2	.4%	195	42.4%	168	36.5%	67	14.6%	28	6.1%
Worry	I worry about getting too much sun on my skin	4	.9%	170	36.9%	190	41.1%	53	11.5%	44	9.5%

*Note*. Four hundred and sixty students answered the question about wearing a hat with a brim. Four hundred and sixty-one of the students answered the other sun protection questions.

**Table 3:** Univariate tests for gender and means and standard deviations for females and males.

Area	Question	*F*	*p*	*η*_*p*_^2^	Female		Male	
					M	SD	M	SD
Sun protection behaviors	I wear sunglasses	5.23	.023	.012	2.59	.88	2.26	.89
	I wear sunscreen	13.96	<.001	.030	2.43	.87	1.99	.72
	I wear hats in the bright sun	4.53	.034	.010	1.77	.82	1.97	.82
	I wear chap stick with sunscreen in it	26.82	<.001	.057	2.56	1.04	1.72	.92
	I wear a hat with a brim on it	9.99	.002	.022	1.71	.87	1.99	.89
Worry	I worry about getting too much sun on my skin	7.89	.005	.017	2.07	1.01	1.69	.81

*Note*. Including “Do not know” answers could have lowered mean scores.

**Table 4:** Univariate tests for parent values about sun exposure and means for student ratings of parent beliefs.

Area	Question	*F*	*p*	*η*_*p*_^2^	Ratings					
					Yes, important to parents		No, not important to parents		Do not know	
					M	SD	M	SD	M	SD
Sun protection behaviors	I wear sunglasses	2.57	.078	.011	2.41^ab^	.81	1.94^a^	.81	2.03^b^	.83
	I wear sunscreen	13.14	<.001	.056	1.91^a^	.84	1.69^a^	.75	1.86	.86
	I wear hats in the bright sun	3.59	.028	.016	2.35^ab^	1.06	2.00^a^	1.04	1.86^b^	1.11
	I wear chap stick with sunscreen in it	4.86	.008	.021	1.89^a^	.92	1.62^a^	.79	1.92	.86
	I wear a hat with a brim on it	4.90	.008	.021	2.10^a^	.97	1.54^a^	.75	1.78	1.00
Worry	I worry about getting too much sun on my skin	13.55	<.001	.057	2.41^ab^	.81	1.94^a^	.81	2.03^b^	.83

*Notes*. ^a^Indicates a significant difference between yes and no answers using Tukey's follow-up tests; ^b^indicates a significant difference between yes and do not know answers using Tukey's follow-up tests. Including “Do not know” answers could have lowered mean scores.
